# Association Between Environmental Factors and Toxigenic *Clostridioides difficile* Carriage at Hospital Admission

**DOI:** 10.1001/jamanetworkopen.2019.19132

**Published:** 2020-01-10

**Authors:** L. Silvia Muñoz-Price, Ryan Hanson, Siddhartha Singh, Ann B. Nattinger, Annie Penlesky, Blake W. Buchan, Nathan A. Ledeboer, Kirsten Beyer, Sima Namin, Yuhong Zhou, Liliana E. Pezzin

**Affiliations:** 1 Division of Infectious Diseases, Department of Medicine, Medical College of Wisconsin, Milwaukee; 2 Collaborative for Healthcare Delivery Science, Medical College of Wisconsin, Milwaukee; 3 Division of General Internal Medicine, Department of Medicine, Medical College of Wisconsin, Milwaukee; 4 Department of Pathology, Medical College of Wisconsin, Milwaukee; 5 Institute for Health and Equity, Medical College of Wisconsin, Milwaukee

## Abstract

**Importance:**

*Clostridioides difficile* infection is the most frequent health care–associated infection in the United States. However, exposure to this organism might occur outside the health care setting.

**Objective:**

To examine whether exposure to environmental factors, such as livestock farms, is associated with a higher probability of being colonized with *C difficile* at hospital admission.

**Design, Setting, and Participants:**

This retrospective cohort study was conducted from May 1, 2017, to June 30, 2018, at a teaching-affiliated hospital in Milwaukee, Wisconsin. All consecutive patients underwent *C difficile* screening using a nucleic acid amplification test at hospital admission. Data analyses were performed from July 2018 to October 2019.

**Exposures:**

The distances from patient residence to the nearest livestock farms, meat processing plants, raw materials services, and sewage facilities were measured in addition to risk factors previously evaluated in other studies.

**Main Outcomes and Measures:**

The main outcome was a positive result on *C difficile* screening tests performed within 72 hours of hospital admission.

**Results:**

A total of 3043 patients admitted to the hospital were included in the final analysis. Of those, 1564 (51.4%) were women and 2074 (68.9%) were white, with a mean (SD) age of 62.0 (15.9) years; 978 patients (32.1%) were admitted to hematology-oncology units. At first admission, 318 patients (10.4%) were detected through testing as *C difficile* carriers. Multivariable logistic regression analyses were performed on a stratified sample of patients based on hematology-oncology admission status. These analyses indicated that although patients admitted to hematology-oncology units were 35% more likely to be colonized with *C difficile*, no significant association existed between their sociodemographic and economic characteristics or health care and environmental exposures and the likelihood of a positive *C difficile* test result. In contrast, among patients admitted to non–hematology-oncology units, comorbidities increased the likelihood for colonization by more than 4 times; women had 60% greater colonization than men, and a history of recent hospitalization (ie, within the preceding 6 months) increased the likelihood of colonization by 70%. Residential proximity to livestock farms were all significantly associated with a higher likelihood of a positive *C difficile* test result. Residential proximity to livestock farms more than doubled the probability of *C difficile* colonization in patients admitted to non–hematology-oncology units.

**Conclusions and Relevance:**

A shorter distance between residence and livestock farms was associated with *C difficile* colonization. Knowledge of the epidemiology of *C difficile* in the community surrounding the hospital is important, as it has potential implications for the incidence of hospital-onset *C difficile* infection.

## Key Points

**Question:**

Is an individual’s location of residence associated with asymptomatic carriage of toxigenic *Clostridiodes difficile*?

**Findings:**

In this cohort study of 3043 adult participants admitted to the hospital, patients who lived closer to livestock farms had a higher likelihood of carrying toxigenic *C difficile* at the time of hospital admission.

**Meaning:**

*Clostridioides difficile *is acquired not only in hospitals but also in the community setting. Livestock farms may expose people to higher loads of *C difficile* via runoff water and to substances that disrupt the intestinal microbiome, placing them at higher risk of *C difficile* colonization.

## Teaser

This cohort study examines whether environmental factors, such as residential proximity to livestock farms, are associated with the probability of toxigenic *Clostridiodes difficile* carriage in patients at the time of hospital admission.

## Introduction

*Clostridioides difficile* infection is the most common health care–associated infection in the United States. In 2015, the Centers for Disease Control and Prevention reported the incidence of *C difficile* infection to be approximately 453 000 cases per year, with an associated annual mortality of 29 300 patients.^1^ Several studies have suggested that some *C difficile* transmission occurs outside of the hospital environment. Among registries that include the incidence of *C difficile* infection, only 65% of cases in the United States and 74% of those in Europe were reported to be associated with the health care environment,^1,2^ suggesting that community exposure may be a factor in the remaining cases. In addition, large studies using whole-genome sequencing of strains that cause *C difficile* infections reported that 45% of those strains were not related to symptomatic cases,^3,4^ suggesting that community factors have a role in *C difficile* acquisition.

Exposure to *C difficile* in the community setting might occur from various sources, including farms, livestock animals, water, and agricultural produce.^5,6,7,8,9,10^ Whole-genome sequencing of *C difficile* strains in humans and animals indicates a bidirectional spread of strains.^11,12^ Previous studies have evaluated factors associated with the presence of *C difficile* colonization at the time of hospitalization.^13,14,15^ However, to our knowledge, environmental exposures to potential sources of transmission have not yet been evaluated. The goal of this study was to identify factors associated with *C difficile* colonization at the time of hospitalization, with a particular focus on demographic characteristics, comorbidities, and proximity to potential environmental exposures, such as livestock farms and meat processing plants. We hypothesized that in addition to comorbidities and recent hospitalizations, environmental exposure to animals would be associated with a greater likelihood of *C difficile* colonization.

## Methods

### Setting and Population

The study was performed at Froedtert Memorial Lutheran Hospital, a 565-bed teaching-affiliated hospital in the Milwaukee, Wisconsin, metropolitan area. In 2016, more than 200 health care–associated laboratory-identified *C difficile* infection cases from this facility were reported to the National Healthcare Safety Network, resulting in a *C difficile* standardized infection ratio of 1.45 (45% more than the facility’s expected number of infections). The 5 units selected for *C difficile* screening were those with the highest rates of *C difficile* infection: 2 hematology-oncology units (66 beds and 18 935 patient-days per year), which included a blood and marrow transplant unit and a solid tumor unit; 1 solid organ transplant unit (27 beds and 7455 patient-days per year); 1 general medical unit (32 beds and 8060 patient-days per year); and 1 intensive care unit (20 beds and 7800 patient-days per year). This study was reviewed and approved by the institutional review board of the Medical College of Wisconsin, and a waiver of informed consent was granted because of the minimal risk associated with the study and because the research could not practicably be carried out without the waiver.

Patients eligible for inclusion were adults who had at least 1 *C difficile* screening test completed within 72 hours after hospital admission between May 1, 2017, and June 30, 2018. Patients with a history of *C difficile* infection within the preceding 6 months were excluded from further analysis. Patients screened for *C difficile* at more than 1 hospital admission were included only once, using the results of the first screening test from the first admission during which the test was performed. Demographic variables, clinical information, and laboratory results were obtained from the electronic health record, and patients’ home addresses were geocoded using ArcGIS software (Esri). This study followed the Strengthening the Reporting of Observational Studies in Epidemiology (STROBE) reporting guidelines for cohort studies.

### *Clostridioides difficile* Testing

All patients underwent *C difficile* screening using a nucleic acid amplification test at hospital admission. Stool samples were collected by nursing staff and sent to the hospital’s clinical laboratory for *C difficile* testing. Rectal swabs were obtained if patients were not able to produce stool samples. Patients were allowed to refuse the administration of rectal swabs, which anecdotally occurred in less than 10% of cases. The *C difficile* screening tests were conducted using the Xpert *C difficile* assay (Cepheid), which targets a conserved region of the cytotoxin B gene, *tcdB*, required for virulence. The test was internally and independently validated for off-label use with rectal swab specimens and formed stools. Formed stools were tested by inserting the swab into or rolling the swab on the surface of the stool specimen until the swab was visibly coated. The swab was then eluted into the Xpert *C difficile* test buffer (provided by the manufacturer) and tested in accordance with the instructions in the product insert.

During the validation, the limit of detection for alternative sources was assessed using swabs coated with stool matrix that had a negative test result for the presence of *C difficile*. Swabs were subsequently submerged in 10-fold dilutions of a *C difficile* suspension (American Type Culture Collection BAA-1875) in normal saline. The limit of detection was found to be approximately 5 × 10^3^ colony-forming units per swab, or 5 × 10^4^ colony-forming units/mL. This limit of detection was 1 log_10_ greater than the limit stated by the manufacturer.

### Measurement

For the purposes of this study, *C difficile* colonization was defined as a positive *C difficile* screening test result obtained within 72 hours of hospital admission. The *C difficile* screening tests were performed as part of a bundle of interventions established by the hospital’s infection control department and were conducted irrespective of a patient’s symptoms. *Clostridioides difficile* colonization was analyzed as a positive or negative dichotomous variable.

We collected information on sociodemographic and economic variables of interest, including age (<50, 50-59, 60-69, 70-79, and ≥80 years), sex, marital status, self-reported race/ethnicity (white, black, Hispanic, and other), number of comorbidities (0, 1-2, 3-5, and ≥6), and neighborhood income. Neighborhood income was measured as the median income of the zip code of the patient’s primary residence based on 5-year estimates from the American Community Survey conducted from 2012 to 2016. Health care exposure was captured by recording the number of admissions and the length of stay (in days) at first hospital admission. Previous hospitalization was defined as an inpatient stay of 3 or more days within the preceding 6 months.

Environmental exposures were measured based on residential distance (in miles) to the nearest business locations of particular interest. Residential addresses were geocoded using the Infogroup database, and businesses of interest were identified using the 6-digit codes from the North American Industry Classification System (NAICS). These businesses included livestock farms (NAICS codes 1121-1124 and 1129), meat processing plants (NAICS code 3116), farm raw materials services (NAICS code 42452), and sewage treatment facilities (NAICS code 22132). Livestock farms included most of the NAICS 112 categories: cattle ranching and farming (NAICS 1121), hog and pig farming (NAICS 1122), poultry and egg production (NAICS 1123), sheep and goat farming (NAICS 1124), and all other animal production (NAICS 1129).^13^ For the analysis, we focused on residential distances to businesses in Wisconsin, Illinois, and Michigan. The geodetic distances (ie, measurements along the earth’s surface) between a patient’s address and the nearest business within each business group were calculated using ArcGIS software.

### Statistical Analysis

Analyses of summary statistics were performed based on the presence of *C difficile* at hospital admission. Multivariable logistic regression models were first applied to the pooled sample to examine the association between the independent variables and *C difficile* colonization at admission. We also evaluated the type of drinking water (ground vs surface) at the zip code level as a potential factor in the likelihood of *C difficile* colonization. These data were obtained from the drinking water system database maintained by the Wisconsin Department of Natural Resources.^16^

After rejecting the post hoc hypothesis of equality of slopes across patients admitted to the hematology-oncology unit compared with those admitted to other units,^14^ we reestimated the multivariable models on the 2 subsamples of patients stratified by unit of admission (hematology-oncology vs other units). All tests were 2-tailed and unpaired, with a significance threshold of *P* = .05. Data analyses were conducted using Stata software, version 15.1 (StataCorp), from July 2018 to October 2019.

## Results

A total of 4617 *C difficile* screening tests were performed at the hospital between May 1, 2017, and June 30, 2018. Of those, 1272 tests were ineligible for analysis because they were duplicates (ie, tests administered during more than 1 hospital admission during the study period; only the test administered at the first admission was included) or were out of the study range. A total of 216 tests were ineligible because the patients tested had a documented *C difficile* infection in the preceding 6 months, and 86 tests were not included because we were unable to geocode the patients’ home addresses (eg, the addresses were post office boxes).

After exclusions, the analytical sample comprised 3043 patients, of whom 318 (10.4%) were colonized with *C difficile* at first hospital admission. The characteristics of the sample, both overall and by *C difficile* colonization status, are shown in Table 1. The mean (SD) age of the cohort was 62.0 (15.9) years, and 1007 patients (33.1%) were 70 years or older at the time of first admission. A total of 1564 patients (51.4%) were women, 2074 (68.9%) were white, and 1451 (47.7%) were unmarried. The median neighborhood income was $51 515 (range, $14 148-$119 512). With respect to comorbid conditions, 1673 patients (55.0%) had 3 or more comorbidities and 341 (11.2%) had no comorbid condition at the time of admission. A total of 723 patients (23.8%) had a hospitalization lasting 3 or more days within the previous 6 months, and 978 patients (32.1%) were admitted to a hematology-oncology unit at their index hospitalization.

**Table 1.** Participant Characteristics, Overall and by *Clostridioides difficile* Colonization Status at First Admission

Characteristic	No. (%)		
	Total	Colonized at Admission	Not Colonized at Admission
No. of participants	3043 (100.0)	318 (10.4)	2725 (89.6)
Age, y			
Mean (SD)	62.0 (15.9)	62.5 (14.6)	61.9 (16.0)
<50	601 (19.8)	50 (15.7)	551 (20.2)
50-59	595 (19.6)	75 (23.6)	520 (19.1)
60-69	840 (27.6)	94 (29.6)	746 (27.4)
70-79	641 (21.1)	61 (19.2)	580 (21.3)
≥80	366 (12.0)	38 (11.9)	328 (12.0)
Female sex	1564 (51.4)	171 (53.8)	1308 (48.0)
Race/ethnicity			
White	2074 (68.9)	214 (67.3)	1860 (68.3)
Black	766 (25.2)	93 (29.2)	673 (24.7)
Hispanic	99 (3.3)	5 (1.6)	94 (3.4)
Other	104 (3.4)	6 (1.9)	98 (3.6)
Married	1451 (47.7)	149 (46.9)	1302 (47.8)
Neighborhood income, median (range), $	51 515 (14 148-119 512)	51 021 (14 148-119 512)	51 848 (14 148-119 512)
No. of comorbidities			
0	341 (11.2)	21 (6.6)	320 (11.8)
1-2	1025 (33.7)	103 (32.4)	922 (33.9)
3-5	1140 (37.5)	131 (41.2)	1009 (37.1)
≥6	533 (17.5)	63 (19.8)	470 (17.3)
Hematology-oncology units	978 (32.1)	124 (39.0)	854 (31.3)
Previous hospitalization	723 (23.8)	106 (33.3)	617 (22.6)
Residential distance to nearest environmental exposure, median (range), miles			
Livestock farm	5.0 (0.1-78.2)	4.5 (0.1-49.2)	5.1 (0.1-78.2)
Meat processing plant	3.9 (0.1-63.3)	4.0 (0.1-39.4)	3.9 (0.1-63.3)
Farm raw materials	13.4 (0.3-104.8)	13.3 (0.3-72.5)	13.4 (0.3-104.8)
Sewage treatment	10.6 (0.2-116.0)	11.3 (0.6-116.0)	10.5 (0.2-100.2)

Summary statistics by *C difficile* colonization status revealed differences across groups by sex, race/ethnicity, number of comorbidities, previous hospitalization, and unit of admission (hematology-oncology vs other units; Table 1). Notably, 106 patients (33.3%) who were colonized with *C difficile* at admission had been hospitalized in the preceding 6 months compared with 617 patients (22.6%) who were not colonized at admission.

Table 2 shows odds ratios (ORs) and CIs from the multivariable logistic regression analyses of characteristics associated with *C difficile* colonization. Among admissions to non–hematology-oncology units, a statistically significant gradient was observed between the number of comorbidities and *C difficile* colonization at admission.

**Table 2.** Participant Characteristics, by Hematology-Oncology Unit Admission Status and *Clostridioides difficile* Colonization Status at First Admission

Characteristic	Admitted to Hematology-Oncology Unit (n = 978)			Not Admitted to Hematology-Oncology Unit (n = 2065)		
	Colonized at Admission, No. (%)	Not Colonized at Admission, No. (%)	OR (95% CI)	Colonized at Admission, No. (%)	Not Colonized at Admission, No. (%)	OR (95% CI)
No. of Participants	124 (12.7)	854 (87.3)	NA	194 (9.4)	1871 (90.6)	NA
Age, y						
Mean (SD)	61.5 (13.0)	63.0 (13.9)	NA	63.2 (15.5)	61.4 (16.8)	NA
<50	16 (12.9)	132 (15.5)	1 [Reference]	34 (17.5)	419 (22.4)	1 [Reference]
50-59	36 (29.0)	164 (19.2)	1.32 (0.63-2.79)	39 (20.1)	356 (19.0)	0.97 (0.48-1.99)
60-69	39 (31.5)	269 (31.5)	0.91 (0.44-1.86)	55 (28.4)	477 (25.5)	1.09 (0.57-2.08)
70-79	23 (18.5)	222 (26.0)	0.55 (0.25-1.22)	38 (19.6)	358 (19.1)	1.22 (0.62-2.37)
≥80	10 (8.1)	67 (7.8)	1.586 (0.62-4.00)	28 (14.4)	261 (13.9)	0.90 (0.41-1.95)
Female sex	58 (46.8)	397 (46.5)	0.96 (0.60-1.52)	113 (58.2)	911 (48.7)	1.59 (1.03-2.46)^a^
Race/ethnicity						
White	97 (78.2)	681 (79.7)	1 [Reference]	117 (60.3)	1179 (63.0)	1 [Reference]
Black	23 (18.5)	118 (13.8)	2.31 (1.01-5.28)^a^	70 (36.1)	555 (29.7)	1.54 (0.83-2.87)
Hispanic	3 (2.4)	26 (3.0)	1.19 (0.24-5.98)	2 (1.0)	68 (3.6)	0.51 (0.07-4.01)
Other	1 (0.8)	29 (3.4)	0.00 (0.00-0.00)	5 (2.6)	69 (3.7)	0.69 (0.16-3.01)
Married	77 (62.1)	517 (60.5)	0.87 (0.53-1.43)	72 (37.1)	785 (42.0)	0.92 (0.59-1.45)
Neighborhood income, median (range), $	60 706 (14 148-119 512)	56 719 (14 148-119 512)	1.00 (1.00-1.00)	45 520 (14 148-119 512)	49 165 (14 148-119 512)	1.00 (1.00-1.00)
Comorbidities, No.						
0	7 (5.6)	70 (8.2)	1 [Reference]	14 (7.2)	250 (13.4)	1 [Reference]
1-2	52 (41.9)	368 (43.1)	2.23 (0.75-6.66)	51 (26.3)	554 (29.7)	4.17 (1.22-14.23)^a^
3-5	54 (43.5)	321 (37.6)	2.11 (0.70-6.36)	77 (39.7)	688 (36.9)	4.97 (1.49-16.62)^a^
≥6	11 (8.9)	95 (11.1)	1.79 (0.49-6.52)	52 (26.8)	375 (20.1)	5.56 (1.61-19.25)^a^
Previous hospitalization	194 (22.7)	37 (29.8)	1.54 (0.92-2.55)	69 (35.6)	423 (22.6)	1.66 (1.06-2.61)^a^
Residential distance to nearest environmental exposure, median (range), miles						
Livestock farm	4.3 (0.2-12.1)	4.8 (0.1-46.3)	0.95 (0.70-1.28)	4.6 (0.1-13.1)	5.2 (0.1-41.4)	0.67 (0.49-0.90)^a^
Meat processing plant	4.4 (0.1-19.6)	4.1 (0.1-43.7)	1.03 (0.79-1.34)	4.0 (0.2-26.0)	3.8 (0.1-37.4)	0.96 (0.74-1.23)
Farm raw materials	13.4 (0.3-22.5)	12.9 (0.3-53.2)	0.97 (0.68-1.39)	13.1 (0.9-22.8)	13.5 (0.3-51.1)	1.08 (0.67-1.73)
Sewage treatment	11.1 (0.6-63.2)	10.6 (0.2-82.3)	0.98 (0.73-1.33)	11.3 (2.9-46.0)	10.4 (0.4-100.2)	1.16 (0.77-1.75)

Abbreviations: NA, not applicable; OR, odds ratio.

^a^ Coefficients are statistically significant at *P* ≤ .05.

The probability of being colonized at admission was also 70% higher among non–hematology-oncology patients who had a history of recent hospitalization (ie, within the preceding 6 months; OR, 1.66; 95% CI, 1.06-2.61), while increased distance between patient residence and the nearest livestock farm was associated with a significant decrease in the probability of colonization (OR, 0.68; 95% CI, 0.49-0.90). Sex was also a risk factor for *C difficile* colonization among patients admitted to units other than hematology-oncology, with women more likely than men to be colonized at admission (OR, 1.60; 95% CI, 1.03-2.46). In contrast, the only factor significantly associated with colonization at the time of admission to a hematology-oncology unit was black race (OR, 2.31; 95% CI, 1.01-5.28). Other individual-level characteristics and environmental or health care–associated exposures, such as number of comorbidities, recent previous hospitalization, or residential distance from livestock farms, did not significantly alter the probability of a positive test result at admission.

To provide an estimate of the relative magnitude of the association between environmental and health care exposures, we calculated adjusted probabilities of *C difficile* colonization for different scenarios of residential distance to livestock farms (1, 10, and 50 miles) and previous hospitalization (yes or no), holding all other factors constant at their original levels based on the multivariable regression coefficients shown in Table 2. The results of these calculations for patients admitted to units other than hematology-oncology (ie, patients for whom such exposures were significantly associated with colonization) are shown in Table 3. No association was found between the type of drinking water and *C difficile* colonization.

**Table 3.** Adjusted Probability of *Clostridioides difficile* Colonization, by Environmental Exposure and Previous Hospitalization Status

Exposure	Adjusted Probability of Colonization, %^**a**^	
	Admitted to Non–Hematology-Oncology Unit With Previous Hospitalization	Admitted to Non–Hematology-Oncology Unit With No Previous Hospitalization
Residential distance to nearest livestock farm, miles		
1	15.7	10.6
10	9.6	6.1
50	6.5	4.0

^a^ Adjusted probabilities were calculated at the individual level assuming different combinations of discrete residential distances to livestock farms and previous hospitalizations, while holding all other factors constant at their original values based on coefficients from the multivariable model shown in Table 2. Individual-level probabilities were then averaged over the entire sample of patients admitted to non–hematology-oncology units.

The independent effect of residential distance to livestock farms was substantial; regardless of health care exposure, the probability of colonization more than doubled for those living 1 mile from a livestock farm compared with those living 50 miles from a livestock farm. Specifically, the probability of colonization increased from 6.5% among those living 50 miles from a livestock farm to 15.7% among those with previous hospitalization and from 4% to 10.6% among those without a recent hospitalization. The increased risk of colonization among patients living 10 miles from livestock farms, a value close to the median distance among our sample, ranged from 48% (risk of colonization increased from 6.5% to 9.6%) among those with previous hospitalization to 52% (risk of colonization increased from 4.0% to 6.1%) among those with no previous hospitalization. A history of recent hospitalization, on the other hand, increased the risk of colonization at admission by 62% (risk of colonization increased from 4.0% to 6.5%) among those with no substantial environmental exposure (ie, those living 50 miles from the nearest livestock farm) and by 48% (risk of colonization increased from 10.6% to 15.7%) among those living 1 mile from such an environmental exposure.

## Discussion

In this study, we found that 1 in 10 patients admitted to 5 units of a large tertiary suburban academic hospital were *C difficile* carriers at the time of admission. Although patients admitted to hematology-oncology units were more likely to be colonized with *C difficile* at admission, with the exception of race, no significant association was observed between patients’ sociodemographic and economic factors or health care and environmental exposures and their likelihood of a positive *C difficile* test result at admission. In contrast, among patients admitted to non–hematology-oncology units, comorbidities, female sex, recent hospitalizations, and closer residential proximity to livestock farms were all associated with a higher likelihood of a positive *C difficile* test result at hospital admission. This finding suggests that among patients whose health is less compromised than those admitted to hematology-oncology units, greater health care and environmental exposures may be factors in the probability of *C difficile* colonization.

Previous studies have examined risk factors for *C difficile* colonization at the time of hospitalization.^15,17,18,19,20^ However, to our knowledge, environmental sources of *C difficile* have not been evaluated. The largest study to evaluate risk factors associated with *C difficile* colonization at the time of hospital admission was performed by Loo et al.^17^ The authors evaluated 4143 patients for *C difficile* colonization using culture methods, finding a colonization rate of 4.4% (less than half the rate observed in our study). Factors associated with *C difficile* colonization included hospitalization in the preceding 12 months, corticosteroid use, previous *C difficile* infection, and the presence of serum antibodies for cytotoxin B. Less than 10% of patients included in the study were receiving chemotherapy or glucocorticoid therapy, reflecting a different patient mix than that of our study, in which more than 30% of patients were admitted to hematology-oncology units. Our study also excluded patients with recent *C difficile* infections.

Leekha et al^18^ evaluated a group of 320 patients, of whom 9.7% had positive *C difficile* test results at admission. The variables associated with *C difficile* colonization included recent hospitalization, long-term dialysis, and corticosteroid use. In another study of 259 patients, of whom 15% were colonized with *C difficile* at hospital admission, recent hospitalization was not found to be associated with colonization,^15^ and exposure to penicillins and cephalosporins was more common in the group with toxigenic *C difficile* colonization. To our knowledge, this is the only study to evaluate race as a factor, finding no association between a patient’s race and *C difficile* colonization.^15^

For decades, *C difficile* was thought to be primarily associated with hospital-related exposures; however, data from studies conducted from 2006 to 2018 suggest that farm animals,^12,21,22,23,24^ pets,^7,25^ fresh produce,^8,10^ retail meat,^26^ and even potable water^9^ are contaminated with *C difficile*. Exposure to such environmental sources of *C difficile* may result in its transmission to humans. Farmers with animals colonized with *C difficile* are at higher risk of being *C difficile* carriers, with identical *C difficile* ribotypes to those of animals.^12^ In 2018, a worldwide collection of *C difficile* strains from both humans and farm animals reported a substantial overlap between *C difficile* strains, indicating bidirectional transmission of *C difficile* from animals to humans and vice versa.^11^ In 2017, Anderson et al^27^ reported that the incidence of community-onset *C difficile* infection (ie, patients who experienced active infection in the community setting) was higher in participants whose residences were nearer to livestock farms and other environmental exposures. Wisconsin has a high number of dairy and pig farms, and these animals have been previously associated with *C difficile* carriage.^11^ One source of *C difficile* colonization in humans is thought to occur by water runoff from farms contaminating the water supply.^28^

Although we found an inverse association between *C difficile* colonization and the distance from a patient’s residence to livestock farms, we did not find similar associations with water treatment plants, meat processing plants, or farm raw material plants. It is important to note that for each type of environmental exposure (ie, livestock plant or water treatment plant), we used the distance to the nearest exposure rather than the number of these exposures in the immediate proximity of the residence. We did not find an association between type of drinking water and *C difficile* colonization, which could in part be owing to the lack of individual-level data for sources of drinking water.

The clinical implications of asymptomatic *C difficile* carriage in the non–hematology-oncology population vary based on geographic location, underlying medical condition (ie, immunosuppression), *C difficile* testing methods, and criteria used for diagnosis of *C difficile* infection. A Danish study evaluated *C difficile* carriage in consecutive patients admitted to 8 medical wards in 2 university hospitals from 2012 to 2013.^29^ Of 3501 patients, 213 (6%) were detected through testing as *C difficile* carriers. The incidence of *C difficile* infection was higher in *C difficile* carriers (20 of 213 patients [ 9%]) than in noncarriers (76 of 3251 patients [2%]; OR, 4.64). A 2015 meta-analysis of asymptomatic *C difficile* colonization evaluated 19 studies, totaling 8725 patients with a pooled prevalence of *C difficile* colonization of 8.1%.^30^ Twenty-two percent of patients colonized with *C difficile* at hospital admission developed *C difficile* infections compared with 3% of patients not colonized at admission (relative risk, 5.86). Thus, better understanding of the epidemiology of *C difficile* colonization is important to tailor preventive measures for *C difficile* infection.

### Limitations

This study has several limitations. Because it was a single-center study, it was limited to the hospital’s geographic catchment area. In addition, we did not explore the variables of outpatient antibiotic exposure, chemotherapy, and medical procedures before hospital admission, as these data points are unreliably documented in the medical records. However, antibiotic and immunosuppressant exposures have been studied as independent variables by other researchers.^15,17,18^ Also, we did not explore the presence of antibodies for *C difficile* toxin B, which was previously reported to be associated with carriage.^17^

## Conclusions

Factors associated with *C difficile* colonization at hospitalization differed based on admission to hematology-oncology units. In addition to previously considered risk factors (eg, previous hospitalization), our analysis indicates that residential proximity to certain environmental exposures, such as livestock farms, is associated with a higher risk of *C difficile* carriage, particularly among the non–hematology-oncology population. This environmental association might be owing not only to higher exposure to *C difficile* but also to exposure to microbiome disruptors, such as antibiotics and pesticides in water runoff.^31^ Future studies should examine the association between *C difficile* and livestock farms in other geographic areas and, if an association exists, identify the underlying mechanisms for this association.
